# Characterization, In Vitro Biological Activity and In Vivo Cardioprotective Properties of *Trametes versicolor* (L.:Fr.) Quél. Heteropolysaccharides in a Rat Model of Metabolic Syndrome

**DOI:** 10.3390/ph16060787

**Published:** 2023-05-25

**Authors:** Marina Nikolic, Nevena Lazarevic, Jovana Novakovic, Nevena Jeremic, Vladimir Jakovljevic, Vladimir Zivkovic, Jovana Bradic, Danijela Pecarski, Gülsen Tel-Çayan, Jasmina Glamocija, Marina Sokovic, Andrej Gregori, Jovana Petrovic

**Affiliations:** 1 Department of Physiology, Faculty of Medical Sciences, University of Kragujevac, Svetozara Markovica 69, 34000 Kragujevac, Serbia; marina.rankovic.95@gmail.com (M.N.); drvladakgbg@yahoo.com (V.J.); vladimirziv@gmail.com (V.Z.); 2 Center of Excellence for Redox Balance Research in Cardiovascular and Metabolic Disorders, 34000 Kragujevac, Serbia; jovana.jeremic@medf.kg.ac.rs (J.N.); nbarudzic@hotmail.com (N.J.); jovanabradickg@gmail.com (J.B.); 3 Department of Pharmacy, Faculty of Medical Sciences, University of Kragujevac, Svetozara Markovica 69, 34000 Kragujevac, Serbia; 4 Department of Human Pathology, I.M. Sechenov First Moscow State Medical University, 119146 Moscow, Russia; 5 I.M. Sechenov First Moscow State Medical University, 119991 Moscow, Russia; 6 Department of Pharmacology of the Institute of Biodesign and Complex System Modelling, I.M. Sechenov First Moscow State Medical University, 119146 Moscow, Russia; 7 The College of Health Science, Academy of Applied Studies Belgrade, 11000 Belgrade, Serbia; danijela.pecarski@assb.edu.rs; 8 Department of Chemistry and Chemical Processing Technologies, Muğla Vocational School, Muğla Sıtkı Koçman University, Muğla 48000, Turkey; gulsentel@mu.edu.tr; 9 Institute for Biological Research “Siniša Stanković”, National Institute of Republic of Serbia, University of Belgrade, Bulevar Despota Stefana 142, 11000 Belgrade, Serbia; jasna@ibiss.bg.ac.rs (J.G.); marina.sokovic@nitra.gov.rs (M.S.); 10 MycoMedica Ltd., Podkoren 72, 4280 Kranjska Gora, Slovenia; andrej.gregori@zanaravo.com

**Keywords:** *Trametes versicolor*, heteropolysaccharides, antioxidant, antimicrobial agent, metabolic syndrome, rat heart, cardioprotection

## Abstract

The present study aimed to examine the biological activity and cardioprotective potential of *Trametes versicolor* heteropolysaccharides (TVH) in a rat model of metabolic syndrome (MetS). This study included 40 *Wistar* rats divided into 5 groups: CTRL—healthy non-treated rats; MetS—non-treated rats; and H-TV, M-TV and L-TV-rats with MetS treated with either 300, 200 or 100 mg/kg TVH per os for 4 weeks. After finishing the treatment, we conducted an oral glucose tolerance test (OGTT), hemodynamic measurements and the animals were sacrificed, hearts isolated and subjected to the Langendorff technique. Blood samples were used for the determination of oxidative stress parameters, lipid status and insulin levels. We showed that α-amylase inhibition was not the mode of TVH antidiabetic action, while TVH showed a moderate inhibition of pathogenic microorganisms’ growth (MIC 8.00 mg·mL^−1^; MBC/MFC 16.00 mg·mL^−1^). H-TV and M-TV significantly reduced the level of prooxidants (O_2_^−^, H_2_O_2_, TBARS; *p* < 0.05), increased antioxidants activity (SOD, CAT, GSH; *p* < 0.05), reduced blood pressure (*p* < 0.05), improved glucose homeostasis in the OGTT test (*p* < 0.05), and ejection fraction (*p* < 0.05) and cardiac contractility (*p* < 0.05) compared to MetS (*p* < 0.05). Moreover, TVH treatment normalized the lipid status and decreased insulin levels compared to MetS rats (*p* < 0.05). The obtained results demonstrated that the TVH may be considered a useful agent for cardioprotection in MetS conditions.

## 1. Introduction

Metabolic syndrome (MetS), also known as syndrome X, can be defined as a disorder including several entities such as insulin resistance, hyperglycemia, hypertension, hyperlipidemia and abdominal obesity according to the WHO [1,2]. At present, this metabolic disorder has become the major health hazard and economic burden of the modern era and society. The prevalence of MetS is higher in western countries and urban populations, due to bad eating habits and sedentary lifestyles. Since MetS most often progresses to type 2 diabetes mellitus (DM2), the incidence of MetS can be assumed based on the incidence of DM2 and obesity. Namely, novel data suggest that over 30 million people age > 18 around globe have DM2, and that almost 25% of them had not been aware of their illness. Thus, the prevalence of MetS is estimated to be at least three times higher [2]. Therapeutic options for MetS management involve changes in lifestyle and the use of antidiabetic and antihyperlipidemic drugs. However, an alternative medicine approach is also gaining popularity due to several advantages, with significantly fewer side effects being the most important one [3].

Recently, more attention has been focused on dietary supplements, nutraceuticals and functional foods that are considered alternative approaches used to mitigate hyperglycemia and prevent the development of MetS and consequent diabetes-related health problems. Studies suggest that natural products are easily absorbed and metabolized by the human body, but also show fewer toxic side effects than conventional medications [4,5]. Among different supplements, several mushrooms stand out as noteworthy products with anti-hyperglycemic, anti-lipidemic, antioxidant and anti-inflammatory activities [6,7,8]. Members of the genus *Trametes*, encompassing approximately 60 different species, are highly appreciated species within the mentioned context. To be more specific, *Trametes versicolor* (L.) Lloyd (hereafter referred to as TV throughout the text), aka *Coriolus versicolor* or turkey tail mushroom, has been the most thoroughly investigated *Trametes* species so far, with a number of publications covering its wide application. Scientific studies and ethno-medicine both indicate that the application of this mushroom may provide several beneficial health effects, which may be attributed to a wide range of bioactive compounds, such as polysaccharides, glycoproteins, glycopeptides and phenolics etc. [9,10,11]. Up to this point, *T. versicolor* has primarily been studied as an anticancer agent. Polysaccharides derived from its fruiting bodies showed immunostimulatory as well as cytotoxic activity towards cancer cells both in vitro and in vivo [12]. Furthermore, the in vitro antioxidant, anti-inflammatory and antimicrobial effects of *T. versicolor* have also been reported. Nonetheless, our literature survey showed a lack of data regarding the glucose homoeostasis regulation and antidiabetic hypolipidemic properties of this mushroom and its polysaccharides [13].

However, to date, no study has ever evaluated the effects of TV heteropolysaccharides (TVH) on cardiac function, especially in animals or patients with MetS. Accordingly, the purpose of this study was to first chemically characterize TVH, and its in vitro antimicrobial and alpha-amylase inhibiting potential, while the main goal was to examine the potentially cardioprotective effects of TVH in vivo in rats with metabolic syndrome. We assumed that a 4-week supplementation of TVH would reduce oxidative stress, ameliorate cardiac function and improve glycemic control in rats with metabolic syndrome.

## 2. Results and Discussion

### 2.1. Chemical Analyses of TV Heteropolysaccharides

#### 2.1.1. FT-IR Spectra

The characteristic functional groups in the TVH were identified by FT-IR spectroscopy. A spectrum was recorded in the frequency range of 4000–400 cm^−1^. As shown in Figure S1 and Table 1, an absorption band at 3273.18 cm^−1^ showed the characteristic stretching vibration of O-H groups. The absorption band at 2915.27 cm^−1^ indicated the -CH stretching vibration, which shows the presence of polysaccharides. The bound water at 1634.65 cm^−1^, -CH-(O-CH_2_) stretching vibrations at 1375.06 cm^−1^ and weak C-O stretching vibrations at 1240.63 cm^−1^ were identified in FT-IR spectrum. The absorption band at 1029.09 cm^−1^ indicated the presence of β-linked glycosyl groups in the main chain.

#### 2.1.2. Monosaccharide Composition 

The results of the GC-MS monosaccharide analysis are presented in Table 2. The predominant monosaccharide identified in TVH was glucose (46.01%), followed by galactose (37.58%), rhamnose (3.34%) and xylose (0.65%). Arabinose, fucose and mannose were not detected in TVH. The GC-MS chromatogram of the monosaccharide standards were given in Figure S2 and the GC-MS chromatograms of monosaccharide composition of TVH in Figure S3.

#### 2.1.3. Molecular Weight

HPLC was used to determine the molecular weight of TVH. The molecular weight of TVH was calculated as 2553.19 Da. The HPLC chromatogram of TVH is shown in Figure S4.

### 2.2. α-Amylase Activity

The obtained results of the enzymatic activity are presented in the Table 3, and they demonstrate that the tested sample has no inhibitory potential towards α-amylase enzyme linked to type-2 diabetes, while positive control acarbose showed this potential.

### 2.3. Antimicrobial Activity

Results of the antimicrobial activity are presented in Table 4. They clearly indicate that TVH has direct antibacterial and anticandidal potential towards tested pathogenic microorganisms isolated from the skin, though it is lower than positive controls.

### 2.4. Effects of TV Supplementation on Hemodynamic Parameters

#### 2.4.1. Blood Pressure Analysis

**Figure 1:**
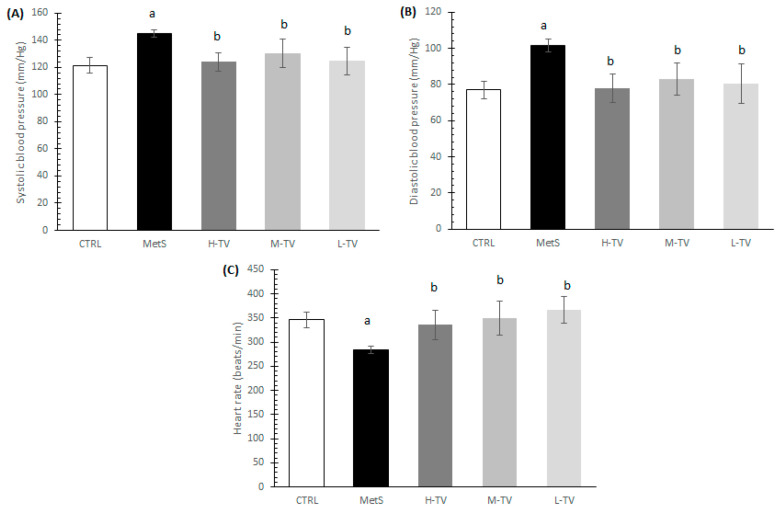
Effects of TVH administration on blood pressure and heart rate. (**A**) Systolic blood pressure (SBP); (**B**) diastolic blood pressure (DBP); (**C**) heart rate (HR). CTRL: control group of healthy non-treated rats; MetS: control group of rats with induced metabolic syndrome; H-TV: rats with metabolic syndrome treated with high dose of *T. versicolor*; M-TV: rats with metabolic syndrome treated with medium dose of *T. versicolor*; L-TV: rats with metabolic syndrome treated with low dose of *T. versicolor.* Data are presented as means ± standard deviation. Data are presented as means ± standard deviation. Statistical significance at the level *p* < 0.05: ^a^ compared to CTRL; ^b^ compared to MetS.

After finishing the 4-week protocol, we evaluated the blood pressure and heart parameters. The induction of MetS led to a significant increase in SBP and DBP and decrease in HR values compared to healthy CTRL animals (*p* < 0.05; Figure 1A–C). Namely, the exposure of animals with MetS to a four-week supplementation of TVH had hypotensive effects. All three applied doses of TVH significantly lowered both SBP and DBP compared to the MetS group without treatment, and these values were similar relative to the CTRL ones. No dose-dependent effects on SBP and DBP were noticed (*p* < 0.05; Figure 1A,B). Regarding the obtained HR value, a significant increment of this parameter was observed in all treated groups (H-TV, M-TV and L-TV) compared to the group of rats with MetS (MetS; *p* < 0.05; Figure 1C).

#### 2.4.2. Echocardiographic Evaluation 

After accomplishing the 4-week protocol, we performed echocardiographic analysis to evaluate the effect of TVH on the dimensions of the left ventricle. It was observed that rats with MetS had significantly lower FS and EF values compared to healthy rats (*p* < 0.05). The supplementation of animals with MetS with high and medium doses of TVH had a significantly improved ejection fraction and fractional shortening compared to the MetS group of rats and compared to the L-TV group (*p* < 0.05). However, significantly lower IVSs and IVSd values were observed in all treated groups compared to the MetS rats, and a similar trend was noticed in LVPWs and LVPWd, and IVSs and IVSd. The most prominent changes in the mentioned parameters were observed in the H-TV and M-TV groups compared to the L-T group (*p* < 0.05; Table 5).

### 2.5. Effects of TVH Supplementation on Systemic Oxidative Stress

**Figure 2:**
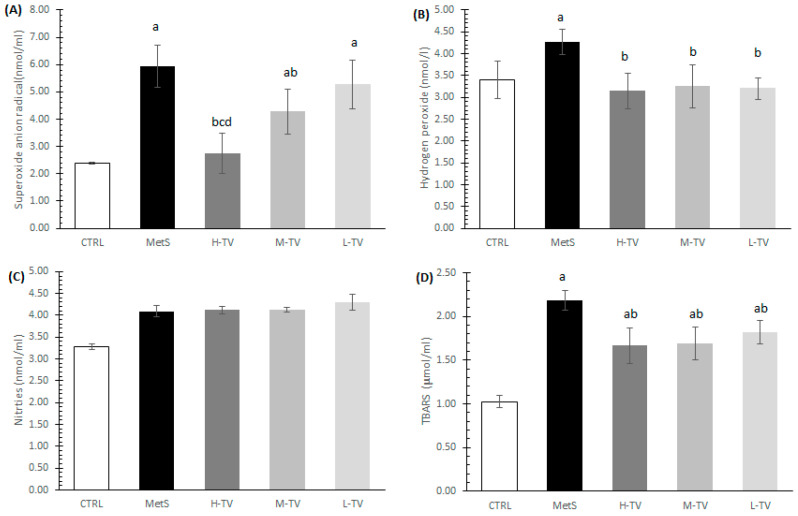
Effects of TVH administration on antioxidant parameters. (**A**) Superoxide anion radical (O_2_^−^), (**B**) hydrogen peroxide (H_2_O_2_); (**C**) nitrites (NO_2_^−^); (**D**) index of lipid peroxidation (TBARS). CTRL: control group of healthy non-treated rats; MetS: control group of rats with induced metabolic syndrome; H-TV: rats with metabolic syndrome treated with high dose of *T. versicolor*; M-TV: rats with metabolic syndrome treated with medium dose of *T. versicolor*; L-TV: rats with metabolic syndrome treated with low dose of *T. versicolor.* Data are presented as means ± standard deviation. Statistical significance at the level *p* < 0.05: ^a^ compared to CTRL; ^b^ compared to MetS; ^c^ compared to H-TV group; ^d^ compared to M-TV group.

The induction of MetS led to a significant increase in the release of prooxidants, O_2_^−^, H_2_O_2_ and TBARS, compared to healthy CTRL rats (*p* < 0.05). The treatment with TVH significantly decreased the release of all measured prooxidant parameters, O_2_^−^, H_2_O_2_ and TBARS, except NO_2_. To be more specific, the levels of both TBARS and H_2_O_2_ were markedly declined in all the MetS groups treated with TVH compared to the non-treated MetS animals (*p* < 0.05), while no dose-dependent effects were noticed (Figure 2B,D). An analysis of the O_2_^−^ release revealed that both high and medium doses of TVH markedly decreased the release of this prooxidant relative to the MetS group (*p* < 0.05), while a low dose was not effective. Additionally, the H-TV group had the most prominent effect on O_2_^−^ compared to medium and low doses of TVH supplementation (*p* < 0.05; Figure 2A).

**Figure 3:**
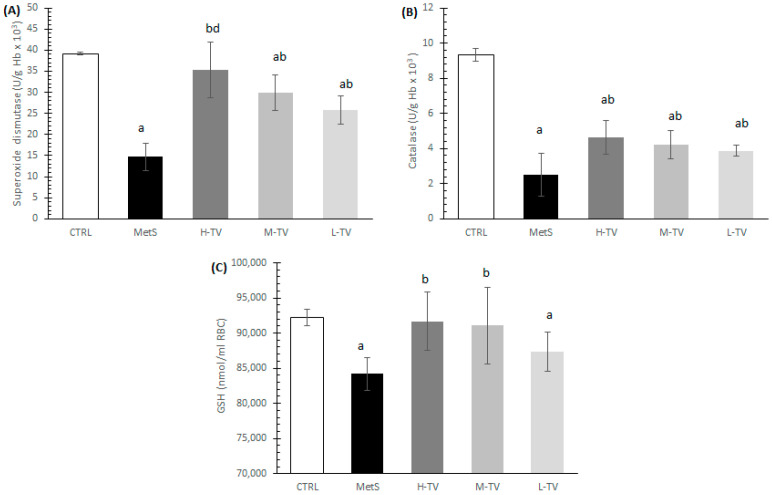
Effects of TVH administration on antioxidant parameters. (**A**) Superoxide dismutase (SOD), (**B**) catalase (CAT) and (**C**) reduced glutathione (GSH). CTRL: control group of healthy non-treated rats; MetS: control group of rats with induced metabolic syndrome; H-TV: rats with metabolic syndrome treated with high dose of *T. versicolor*; M-TV: rats with metabolic syndrome treated with medium dose of *T. versicolor*; L-TV: rats with metabolic syndrome treated with low dose of *T. versicolor.* Data are presented as means ± standard deviation. Statistical significance at the level *p* < 0.05: ^a^ compared to CTRL; ^b^ compared to MetS; ^d^ compared to L-TV group.

The rats with MetS showed signs of highly compromised antioxidant systems; their levels of SOD, CAT and GSH were significantly lower than in control group of healthy individuals (*p* < 0.05). The four-week administration of TVH in all three investigated doses induced a significant increment of antioxidant enzymes activity (SOD and CAT) compared to the MetS rats. The increase in SOD activity in the H-TV group was superior relative to the L-TV group (*p* < 0.05), while there were no significant differences in CAT activity between the three applied doses of TV (*p* > 0.05; Figure 3A,B). As for the reduced GSH form level, this was significantly increased in the groups treated with high and medium doses of TVH in comparison with the non-treated MetS rats (*p* < 0.05), while a low dose of TVH did not improve GSH level compared to the MetS (*p* > 0.05; Figure 3C).

### 2.6. Effects of TVH Supplementation on OGTT

**Figure 4:**
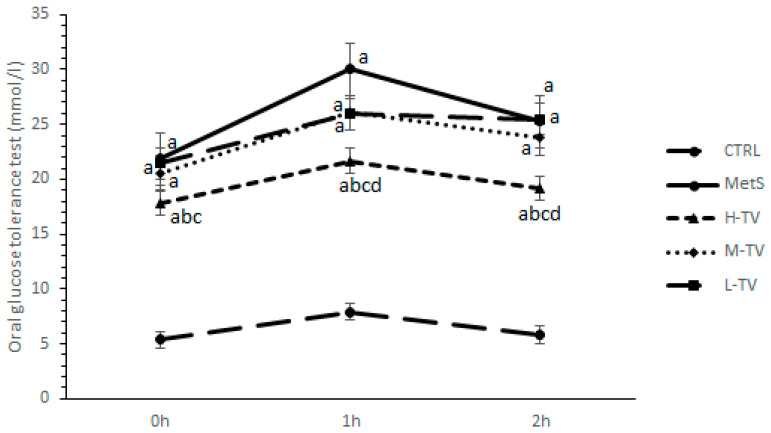
Effects of TVH administration on oral glucose tolerance test (OGTT). CTRL: control group of healthy non-treated rats; MetS: control group of rats with induced metabolic syndrome; H-TV: rats with metabolic syndrome treated with high dose of *T. versicolor*; M-TV: rats with metabolic syndrome treated with medium dose of *T. versicolor*; L-TV: rats with metabolic syndrome treated with low dose of *T. versicolor.* Data are presented as means ± standard deviation. Statistical significance at the level *p* < 0.05: ^a^ compared to CTRL; ^b^ compared to MetS; ^c^ compared to M-TV group; ^d^ compared to L-TV group.

An oral glucose tolerance test (OGTT) was performed on the last day of the experiment. The obtained results showed that the fasting glucose level (0 h) significantly dropped in a group of rats treated with the highest dose of TVH extract compared to the MetS rats and M-TV group (*p* < 0.05), while no differences were observed between the MetS, L-TV and M-TV. One hour post-glucose loading, a supplementation of high-dose TVH significantly decreased the glucose level compared to all other groups (MetS, M-TV and L-TV; *p* < 0.05). Similar results, including lower values of glycaemia in the H-TV group compared to the other investigated groups (*p* < 0.05; Figure 4), were also observed in the second hour of measurement. However, none of the TVH doses succeeded in reaching glucose values similar to those in the CTRL group.

### 2.7. Insulin Level Measurement

**Figure 5:**
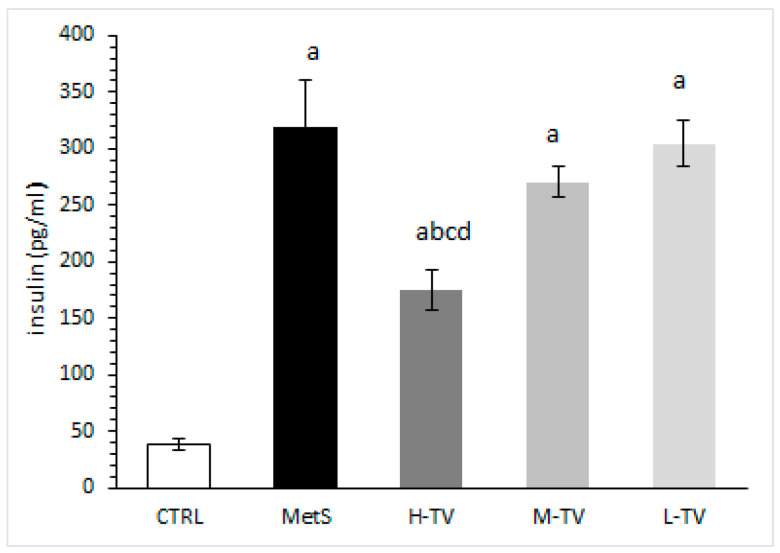
Effects of TVH supplementation on serum insulin level. CTRL: control group of healthy non-treated rats; MetS: control group of rats with induced metabolic syndrome; H-TV: rats with metabolic syndrome treated with high dose of *T. versicolor*; M-TV: rats with metabolic syndrome treated with medium dose of *T. versicolor*; L-TV: rats with metabolic syndrome treated with low dose of *T. versicolor*. Data are presented as means ± standard deviation. Statistical significance at the level *p* < 0.05: ^a^ compared to CTRL; ^b^ compared to MetS; ^c^ compared to M-TV group; ^d^ compared to L-TV group.

Our results showed that the serum insulin level was significantly increased in the MetS rats, compared to the healthy CTRL rats (*p* < 0.05). However, only the highest dose of TVH succeeded in markedly lowering this parameter compared to the MetS group, and the groups treated with low and medium TVH doses (*p* < 0.05). Additionally, none of the applied TVH doses decreased insulin levels to control values (Figure 5).

### 2.8. Lipid Status

**Figure 6:**
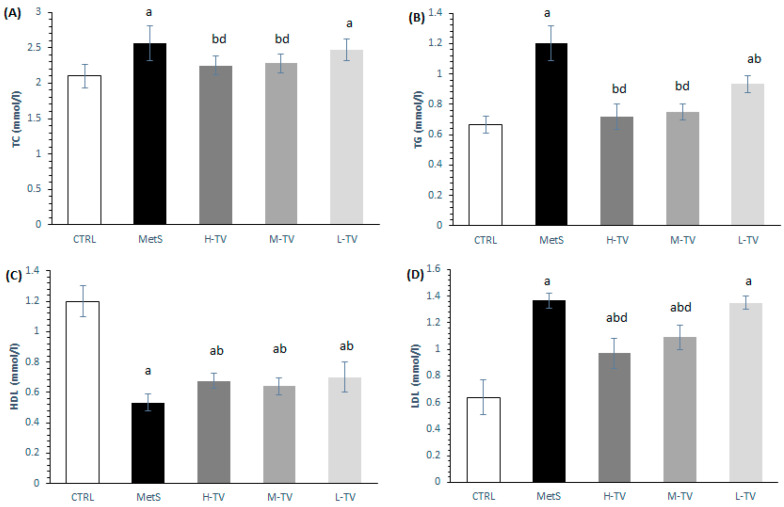
Effects of TVH supplementation on lipid parameters: (**A**) total cholesterol—TC; (**B**) triglycerides—TG; (**C**) high density lipoprotein (HDL) and (**D**) low density lipoprotein (LDL). CTRL: control group of healthy non-treated rats; MetS: control group of rats with induced metabolic syndrome; H-TV: rats with metabolic syndrome treated with high dose of *T. versicolor*; M-TV: rats with metabolic syndrome treated with medium dose of *T. versicolor*; L-TV: rats with metabolic syndrome treated with low dose of *T. versicolor*. Data are presented as means ± standard deviation. Statistical significance at the level *p* < 0.05: ^a^ compared to CTRL; ^b^ compared to MetS; ^c^ compared to M-TV group; ^d^ compared to L-TV group.

The basic parameters of the lipid panel were measured from serum samples collected at the end of the experimental period. The induction of MetS led to a significant increase in TC, TG and LDL levels coupled with a drop in HDL levels compared to the CTRL rats (*p* < 0.05; Figure 6). However, a supplementation with TVH in all the tested doses succeeded in decreasing TG and increasing HDL levels compared to the untreated MetS rats (Figure 6B,C). While no dose-dependent effect was detected in the HDL values, the highest and medium doses of TVH led to more prominent changes in the TG levels compared to the L-TV group (Figure 6B). The same trend was observed in the TC and LDL values (Figure 6A,D). Although rats from the H-TV and M-TV groups had significantly lower levels of LDL compared to the MetS (*p* < 0.05; Figure 6D), a supplementation with the highest and medium doses of TVH did not succeed in reaching the value found in the CTRL rats. 

### 2.9. Ex Vivo Cardiac Function

**Figure 7:**
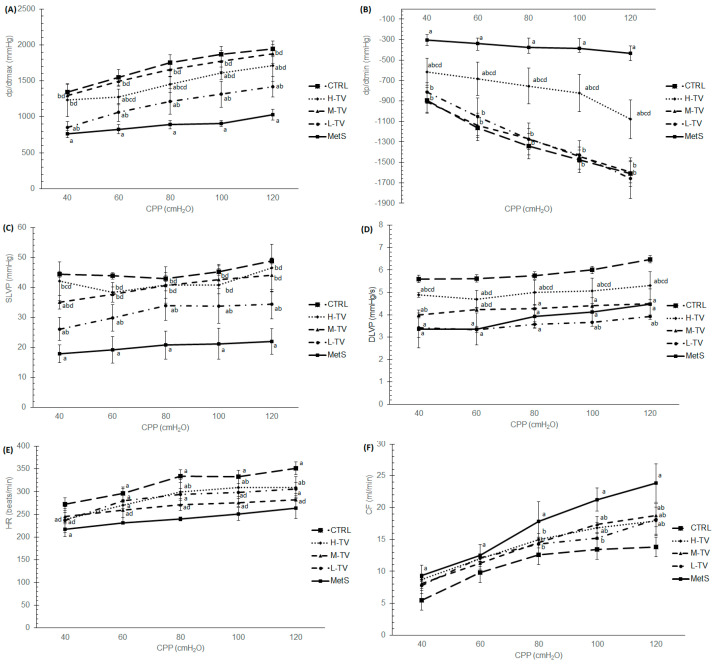
Effects of TVH extract administration on cardiodynamic parameters: (**A**) maximum rate of left ventricular pressure development − dp/dt max; (**B**) minimum rate of left ventricular pressure development − dp/dt min; (**C**) systolic left ventricular pressure – SLVP; (**D**) diastolic left ventricular pressure − DLVP; (**E**) heart rate – HR; (**F**) coronary flow − CF. CTRL: control group of healthy non-treated rats; MetS: control group of rats with induced metabolic syndrome; H-TV: rats with metabolic syndrome treated with high dose of *T. versicolor*; M-TV: rats with metabolic syndrome treated with medium dose of *T. versicolor*; L-TV: rats with metabolic syndrome treated with low dose of *T. versicolor.* Data are presented as means ± standard deviation. Statistical significance at the level *p* < 0.05: ^a^ compared to CTRL; ^b^ compared to MetS; ^c^ compared to M-TV group; ^d^ compared to L-TV group.

The results obtained in this study clearly demonstrate that all the examined cardiodynamic parameters were significantly altered in the group of untreated MetS rats compared to the healthy control group (*p* < 0.05; Figure 7). Major changes in both the dp/dtmax and dp/dtmin parameters were registered in the M-TV group compared to the MetS rats (*p* < 0.05; Figure 7A,B). The values of the dp/dtmax parameter of contractility were markedly elevated in all treatment regimens, with medium and high doses of TVH extract being more effective than low doses at all CPPs (*p* < 0.05; Figure 7A). As for dp/dt min, medium and low doses of TVH achieved more prominent changes compared to the H-TV group (*p* < 0.05; Figure 7B). The most important result obtained within the ex vivo cardiac function was that medium doses of *T. versicolor* were able to reach rather similar values of dp/dt max and dp/dt min at all CPPs to those obtained in the CTRL group of rats (Figure 7A,B), while the dp/dt min value in rats treated with the low dose of TVH reached the CTRL values only at higher pressures (100–120 cm H_2_O) (Figure 5B). As for the SLVP, the results demonstrate that its level was significantly elevated in all three applied dose regimens of TVH supplementation compared to the non-treated MetS group, at all CPPs (*p* < 0.05). However, high and medium doses of TVH induced more a noticeable increment of SLVP compared to the L-TV group (*p* < 0.05; Figure 7C). Moreover, at higher CPPs (80–120 cm H_2_O), approximately similar SLVP values were obtained after treatment with medium and high doses of TVH compared to the CTRL group of rats (Figure 7C). On the other hand, DLVP was mostly affected by the high dose of TVH compared to both the MetS and L-TV groups at all CPPs (*p* < 0.05) but, ultimately, it did not reach the CTRL value. On the other hand, medium doses increased DLVP only at lower CPPs compared to the MetS (40 and 60 cm H_2_O). The treatment with low doses of the extract did not significantly change DLVP level compared to the MetS (*p* > 0.05; Figure 7D).

The treatment with TVH remarkably increased HR at all pressures except 40 cmH_2_O compared to the non-treated MetS rats, while the most prominent effect was achieved in the H-TV group, and especially at 80 and 100 cmH_2_O, almost reaching the values in the CTRL group (*p* < 0.05; Figure 7E). The values of CF in the CTRL group were significantly decreased at all CPPs compared to the MetS rats (*p* < 0.05; Figure 7F). However, there were no significant differences observed in the CF values at the lower CPPs (40–60 cmH_2_O) between the TVH-treated group and the rats with MetS. On the other hand, at higher pressures (80–120 cmH_2_O), the MetS hearts showed highest CF values, while supplementation with TVH in all three applied doses markedly reduced CF compared with the MetS group (*p* < 0.05), with no dose-dependent effect noticed (Figure 7F). 

## 3. Discussion

Data in the literature suggest that the precise chemical characterization of sugars is absolutely necessary, since their bioactive potential is strongly influenced by the mono- and polysaccharide composition, viscosity, solubility, molecular weight and advanced structure [14]. Techniques such as monosaccharide composition by GC-MS and molecular weight by HPLC and FT-IR are widely used for the chemical analysis of mushroom sugars. The most commonly reported monosaccharides of mushrooms include glucose, mannose, xylose, galactose, rhamnose, arabinose and fucose. Less frequently, ribose, fructose, glucuronic acid, N-acetylglucosamine, galacturonic acid and N-acetyl-galactosamine are identified [14]. In the present study, the predominant monosaccharides were glucose and galactose. This is in accordance with previous studies which reported that glucose was the most abundant monosaccharide composition in the polysaccharide of *T. versicolor* as well [15,16,17]. The FT-IR spectrum of TVH by Kozarski et al. showed the characteristic absorption bands at 3425.1 cm^−1^ stretching vibration O–H, 2921 cm^−1^-CH stretching vibration, 1411 cm^−1^ stretching vibration of aliphatic C–C chain, 1023.8 cm^−1^ stretching vibration of C–O and 890.1 cm^−1^ stretching vibration of β-glycosidic linkage [18]. Similar FT-IR results of polysaccharides from *T. versicolor* were reported by other authors as well [14,16,17,19]. A recent study explored the molecular weight of polysaccharides from *T. versicolor* and revealed the presence of exopolysaccharide (EPS) with a molecular weight of 2.45 × 10^4^ Da [14]. A protein-bound polysaccharide from *Coriolus versicolor* with a molecular weight of ~2 × 10^6^ Da was isolated by Cui et al. (2007) [20], whereas Jhan et al. reported *T. versicolor* polysaccharopeptides with 300, 190, 140 and 50 kDa molecular weights [19]. In a different study by Huang et al., the intracellular (IPTV) and extracellular polysaccharide extracts of *T. versicolor* (EPTV) were obtained with molecular masses of 127 and 68.4 kDa, respectively [16]. Two polysaccharides, CVPn and CVPa, were isolated from *Coriolus versicolor* after water extraction and ethanol precipitation by high-performance gel permeation chromatography (HPGPC) analysis. Two isolated polysaccharides with molecular weights of 50.8 kDa for CVPa and 29.7 kDa for CVPn both consisted of the (l→4)-β-/(1→3)-β-D-glucopyranosyl group as a branch-linked backbone at the O-6 site [17]. The obtained data are in full agreement with the previous studies on the characterization of *T. versicolor* polysaccharides.

Regarding the in vitro α-amylase inhibitory activity of TVH, this assay was used as a pharmacological tool for targeting possible compounds which may delay or inhibit postprandial hyperglycemia, thus affecting MetS management. Many natural compounds turned out to have exquisite inhibitory potential regarding this enzyme, which makes them good candidates for developing bioactive agents that alleviate the risk of developing MetS. However, our results indicate that TVH does not show antidiabetic potential in vitro measured through this assay, while the positive control acarbose exerted α-amylase inhibitory effects (Table 3). Since TVH showed extraordinary potential in vivo in MetS, the results we obtained regarding in vitro activity raises questions with respect to the TVH mode of action, which, as it seems, does not include an α-amylase inhibitory activity. Previously published data provide a possible explanation, since several other anti-hyperglycemic mechanisms of mushroom polysaccharides have also been involved in this matter. According to Arambasic et al., mushroom polysaccharides achieve antidiabetic activity via: (1) the inhibition of glucose absorption, (2) the enhancement of pancreatic β-cell mass, as well as (3) an increase in insulin signalling. All these mechanisms, as well as kinetics, should be further investigated in more detail in order to use TVH as proper medicines [21]. Furthermore, it has been also demonstrated that α-amylase inhibitory activity may be ascribed to a major group of polyphenolic compounds, which provides yet another explanation for the results we obtained for TVH [22]. 

Until recently, various skin conditions were not associated with the diagnosis of MetS in patients suffering from it. However, it has become clear that these complex multisystemic disorders may very well be dependent on each other [23]. Due to a damaged skin barrier caused by continuous inflammation and changes in the vasodilation system of the skin provoked by the MetS, superficial parts of the skin become exposed and open for the colonization of pathogenic microorganisms. Bearing in mind that people suffering from MetS have an impaired immune response, they may be more susceptible to the serious course of otherwise non-life-threatening microbial infections. Hence, the antimicrobial effect of TVH on skin clinical isolates was evaluated, to determine whether the application of polysaccharides may inhibit the growth of commonly isolated skin pathogenic microorganisms. Our results indicate that applied concentrations of TVH were able to moderately and uniformly inhibit the growth of all the tested microorganisms except *S. lungdunensis* with MIC values of 8.00 mg/mL and MBC/MFC values of 16.00 mg/mL (Table 4). Although the obtained bactericidal/fungicidal concentrations seem higher than usual for the antimicrobial activity of mushrooms, one should keep in mind that the direct antimicrobial activity of polysaccharides is not usual. Namely, polysaccharides most often realize their antimicrobial potential through an improvement in immune functions, thereby affecting the overall defense ability of the organism. This was demonstrated, for example, in a study conducted by Shi et al.; β-glucans from *C. versicolor* exert antiviral properties on influenza in experimental in vivo systems, via an increase in the production of various cytokines [24]. No direct antimicrobial effects were demonstrated with *C. versicolor* polysaccharides, but a boost in the phagocytic activity of macrophages was clear, suggesting an indirect increase in defensive mechanisms.

Mushrooms as mycochemicals are being widely studied since they have a dual role, acting both as important and valuable food but also as a source of biologically active substances. The most important physiologically active compounds for the biological activity of mushrooms are glucans, considered biological response modifiers. Much research has been oriented towards discovering the properties of glucans, indicating its ability to act both as a drug or an adjuvant therapy in different diseases [25]. With respect to the aim of this study, several mushroom species were confirmed to possess hypoglycemic properties, including *T. versicolor* [26]. Along with these results, the most recent data undoubtedly showed that the administration of a *T. versicolor* fruiting body in vivo may be of assistance in keeping glucose metabolism under control, but that it can also maintain a balance in blood lipid levels, with these beneficial health effects accomplished through the activation of antioxidant enzymes [11,26,27]. The results obtained in this study are in accordance with previously published data, however, they encompass a rather wide range of health issues related to MetS, making them highly applicable in developing novel strategies for the management of this disorder. Herein, we used a MetS animal model and one of the goals was to also evaluate whether a 4-week usage of TVH regulates blood glucose homeostasis by using an OGTT test. Our results showed that a high dose of TVH successfully regulated blood glucose levels compared to the MetS group without treatment in all observed time points. These results are consistent with those obtained by Meng et al., where supplementation with TVH significantly reduced blood glucose levels in diabetic mice at 0 h, 90 min and 120 min time points during an OGTT test, especially regarding high doses [11]. Additionally, similar results were confirmed in diabetic rats, where the extracellular polysaccharopeptides, isolated from TV, showed a dose-dependent effect on fasting glucose levels, an area under the curve in an OGTT test and insulin resistance indices [28]. Among the isolated polysaccharides from *T. versicolor*, the most important one is krestin, which, per se, has been proved to exert hypolipemic, insulin-resistance-ameliorating and hypoglycemic effects, with the regulation of cytokine expression as the possible mechanism of these actions [29]. The previously mentioned bioactive properties of mushroom polysaccharides are highly dependent on their molecular weight, monosaccharide content, branching pattern, glycosidic bond type and substitution type. Taking this into account, some authors highlighted the importance of the presence of the (1→4) glycosidic linkages for the *α*-glucosidase-inhibiting potential of the polysaccharide from *T. versicolor* [30]. The other objective of our study, which evaluated the hypolipidemic role of TVH supplementation in rats with blood-lipid metabolic disorder induced by a high-fat diet, showed that dietary supplementation with TVH for 4 weeks normalized elevated levels of TG, TC and LDL, particularly in the highest and medium doses. However, neither dose regimen was able to reach the HDL values found in the serum of healthy untreated rats, possibly due to the inability of TC to transfer from peripheral tissues to the liver using reverse cholesterol transport pathways. The findings obtained in this study correlate with the recently published research indicating a two-fold stronger hypolipidemic effect of *T. versicolor* supplementation of rats fed with a high-fat diet compared to simvastatin, especially in doses of 100 mg/kg/d and 200 mg/kg/d, respectively [25]. The cardiovascular complications of diabetes and MetS are very common and may seriously endanger the health of these patients, thus, natural products are gaining popularity as adjuvant therapies in DM or as sources of new both hypoglycemic and cardioprotective drugs, due to their safety and effectiveness. Hence, our goal to examine the cardiovascular effects, such as blood-pressure-lowering potential and both in vivo and ex vivo cardiac function, showed an increased potential medicinal value of TVH. In addition to this, the oxidative stress that is often correlated with MetS pathogenesis was ameliorated by TVH consumption, which may be considered one of the potential mechanisms of cardioprotection.

There is insufficient data regarding the in vivo cardiovascular effects of *T. versicolor* in the literature, however, up until now, it is known that several medicinal mushrooms may act as antihypertensives and may preserve EF in diabetic conditions and protect the heart [31,32]. In that sense, the hypotensive effects of *Ganoderma lucidum* have been confirmed and linked with its polysaccharide components, thus we might hypothesize that the TVH we investigated exerted hypotensive effects in a similar manner via an inhibition of the angiotensin converting enzyme (ACE) [32]. Moreover, clinical evidence regarding the cardioprotective effects of polysaccharide fractions from *G. lucidum* in patients with coronary heart disease demonstrates a decrease in diastolic blood pressure, in the percentage of abnormal ECG, as well as in overall cardiac symptoms (chest pain, palpitation, angina pectoris and shortness of breath) [33]. Thus, the achieved cardioprotective effects of TVH confirmed in this study may be related to its polysaccharide fraction, mostly made of glucose, galactose and rhamnose-linked heteropolysaccharides.

Another goal was to assess the in vivo cardiac function after a 4-week supplementation with TVH and our results indicate the superior effect of high and medium doses of TVH in improving EF and FS in MetS conditions compared to the low-dose treatment and non-treated animals. It was recently shown that a 12-week treatment with aqueous *T. versicolor* extract effectively alleviated cardiac dysfunction in a type 1 diabetes mellitus (DM1) rat model, via increasing EF and FS, compared to DM1 rats and restored it to the control values of healthy rats, which is in accordance with our results. The authors highlighted that TV extract could markedly reduce cardiac fibrosis in diabetic rats, mediated by the inhibition of transforming growth factor β1(TGF-β1)/Smad signal transduction, and also decrease cardiac inflammation in diabetes, thus preventing diabetic cardiomyopathy. However, a different treatment regimen was used and a much lower dose of TV extract (25 mg/kg) compared to present study [34]. Nonetheless, to our knowledge, there are no other additional available studies dealing with a similar pathology and methodology with which to compare. It is proved that medicinal mushrooms, such as *G. lucidum*, may improve EF in a rat model of doxorubicin-induced cardiomyopathy and TAC mice via anti-inflammatory and antiapoptotic mechanisms [35,36].

We also wanted to investigate the effect of the applied treatment with TVH on the isolated heart function during the ex vivo retrograde perfusion of the heart. Our results clearly showed that the contractility properties of the heart, estimated by dp/dt max and dp/dt min, were improved at all CPPs in the treated groups, with the most prominent effect in the M-TV group, showing the ability of TVH to preserve cardiac contractility in MetS in both hypoxic, normoxic and hyperoxic conditions. Furthermore, both SLVP and DLVP were positively affected by TVH supplementation. The HR value was also increased in the treated groups with TVH at normoxic and hyperoxic conditions, which is positively correlated with the increased HR measured in vivo. These observations confirm the assumption and hypothesis that TVH may act as a cardioprotective, while the achieved effects may be mediated by antioxidant, hypoglycemic, hypotensive, anti-inflammatory and apoptotic properties.

The results of our study confirmed in vivo that a 3-week consumption of TVH acts as an antioxidant via achieving a decline in the release of free radicals O_2_^−^ and H_2_O_2_, and reducing TBARS, in MetS conditions. In addition, we showed that TVH may improve the antioxidant protection system by increasing SOD and CAT activity and GSH level. The antioxidant properties of *T. versicolor* have been confirmed in several studies, however, mostly by using in vitro methods [37,38,39,40,41]. Two studies conducted by Janjusevic et al. revealed that *T. versicolor* is a potential source of antiradical agents, and highlighted the superior ability of aqueous TVH extract to neutralize DPPH^•^ and ^•^OH radicals compared to ethanolic and methanolic TVH extracts. Additionally, in their second study, a NO^•^ neutralizing activity was confirmed. All of these antioxidant effects could be ascribed to the antioxidant properties of the phenolics and flavonoids present in the TVH extract, especially p-hydroxybenzoic and gallic acid, as well as the flavonoids quercetin, isorhamnetin and catechin. Similar to our observations, another study conducted in diabetic mice confirmed the improvement in SOD activity and glutathione peroxidase (GpX), and the reduction in malondialdehyde (MDA), a product of lipid peroxidation. This was achieved after a four-week supplementation with *T. versicolor* in medium and high doses (2000 and 4000 mg/kg), however, the doses used in the mentioned study were much higher than those that we used [11]. The significant decrease in O_2_^−^ release observed after the TVH administration that we observed may be explained by the fact that increased SOD activity was used to neutralize it. Another author suggested the antioxidant activity of the isolated extracellular polysaccharopeptides from TVH in DM2 rats after 4-week consumption, as shown by the increase in SOD activity and GSH level and decrease in lipid peroxidation products level. These results correlate with our findings, suggesting that the amelioration of oxidative stress by TVH in MetS conditions may also originate from polysaccharopeptides, besides the phenolic acids and flavonoids mentioned [28].

For the first time, the results obtained in this study highlighted the cardioprotective potential of *T. versicolor* heteroploysaccharides both in vivo and ex vivo in the treatment of MetS. This was mediated by the lowering blood pressure effects and favorable effects on heart function, together with the hypoglycemic and oxidative-stress-reducing properties. The promising role of this mushroom in the management of MetS-related diseases is suggested. This study may be a starting point for additional preclinical and clinical research which would fully evaluate the effects and elucidate the exact mechanisms of cardioprotection by *T. versicolor* in MetS conditions. Since the toxicity of *T. versicolor*, even in increased doses (5 g/kg) and prolonged consumption, has not yet been regarded as a matter of concern, the wide exploitation of the mushroom should be suggested [42]. A previous study reported that commercially available TVH preparations can be used as chemoimmunotherapy agents in cancer treatment [43]. In addition to safe oral consumption, a novel TV strain showed great potential in the regulation of glucose homeostasis due to its high level of extracellular polysaccharopeptides. Moreover, this beneficial effect is believed to be associated with an increased relative percentage of D-glucose and D-galactose in polysaccharides, which is closely related to the intracellular polysaccharides that have alpha-glucosidase inhibitory effects [44].

## 4. Materials and Methods

### 4.1. Preparation of Mushroom Heteropolysaccharides

Organic cellulose and supplements containing vegan, gluten- and allergen-free substrate with moisture content of 65% (*w*/*w*) were filled into each polypropylene bag (3 kg) with breathing filters and sterilized for 80 min at 121 °C. After sterilization, substrate was cooled to room temperature and inoculated with TV culture obtained from fungal culture bank of MycoMedica Ltd., Podkoren, Slovenia. Culture was cultivated in dark conditions on potato dextrose agar at 24 °C and, when fully overgrown, dispersed into one liter of sterilized water using a Waring blender (PA, USA). After dispersion, fungal culture was poured onto the substrate and incubated at 24 °C. After approximately three months of cultivation, fungi were harvested. A part of the material was extracted, mixed with the first part and dried under vacuum for 24 h at temperatures below 60 °C with the addition of a drying agent—organic gluten-free starch. After drying, the material, i.e., TVH (sold under commercial brand named “GOBA *Trametes versicolor*”), contained moisture content lower than 7%. Product was milled into a fine powder with majority of particles smaller than (250 µm) and used for further analysis. Production batch TRVE26052021 of TVH was used in this study, a sample of which is deposited in production batch collection at Mycomedica Ltd., Podkoren, Slovenia.

### 4.2. Chemical Analyses

#### 4.2.1. FT-IR Analysis

The infrared spectrum of the TVH was determined using a Fourier transform infrared spectrophotometer (Thermo Scientific one Nicolet IS10, MA, USA). Spectrum was recorded for FT-IR measurement in the frequency range 4000–400 cm^−1^.

#### 4.2.2. Monosaccharide Composition

The monosaccharide composition of TVH was determined by GC/MS (Varian Saturn 2100T, IL, USA) according to method [45] with modifications [46]. TVH (10 mg) was hydrolysed with 0.1 M trifluoroacetic acid (2 mL) for 3h at 110 °C, after which the methanol was added to remove TFA (trifluoroacetic acid), and resulting mixture was evaporated using a rotary vacuum evaporator at 45 °C. The hydrolysate was converted to its trimethyl silyl derivatives after addition of N, O-bis(trimethylsilyl) trifluoroacetamide (BSTFA, 300 µL) and pyridine (200 µL), after which the resulting mixture was heated at 80 °C for 30 min. Standard sugars (arabinose, rhamnose, fucose, xylose, mannose, galactose and glucose) were converted to their TMS (trimethylsilyl) derivatives in the same manner. Sample and standard derivatives were injected into GC/MS (Varian Saturn 2100T, USA) equipped with HP-5 fused silica capillary column (30 m × 0.32 mm × 0.25 mm). The following chromatographic conditions were used: He was used as the carrier gas at a flow rate of 1 mL/min; the temperature of injector and detector were set at 250 °C and 270 °C, respectively; initial column temperature was maintained at 80 °C for 3 min, after which it was increased gradually to 280 °C at a rate of 10 °C/min and held at 280 °C for 10 min. Monosaccharide compositions were identified by comparison with seven standard sugars. According to the chromatogram, the relative molar ratios of monosaccharides were analysed by the area normalization method.

#### 4.2.3. Molecular Weight Determination

Molecular weight of the TVH was determined using HPLC Shimadzu LC-20 AT equipped with a GPC Ultrahydrogel 1000 (7.5 mm × 300 mm) column and a Shimadzu RID-10A detector set at 40 °C (Shimadzu, Tokyo, Japan) according to the method previously described [47], with slight modification [46]. Sample solution of 20 μL was injected in each run, and eluted with 0.05 M NaCl at a flow rate of 0.5 mL/min. Standard dextrans (1000, 5000, 12,000, 25,000, 50,000, 80,000, 150,000, 270,000, 410,000 and 670,000 Da) were passed through the calibrated column, after which the elution volumes were plotted against the logarithm of their respective molecular weights. The elution volume of the purified polysaccharide was plotted in the same graph, and the molecular weight was determined. The molecular weight of the TVH was calculated according to equation obtained from the standard dextran graph:

Elution volume = −1.2764[logMW] + 15.168 (*r^2^*: 0.970)

### 4.3. α-Amylase Inhibition Assay

Enzymatic assay was performed as previously described [48]. Various concentrations of TVH (500 μL) were mixed with 0.02 M sodium phosphate buffer (pH 6.9 with 0.006 M NaCl, 500 μL) containing Porcine pancreatic α-amylase (Sigma Aldrich, Darmstadt, Germany) (0.5 mg/mL). The resulting mixture was incubated for 10 min at 25 °C. Subsequently, 1% starch solution (500 μL) in 0.02 M sodium phosphate buffer (pH 6.9 with 0.006 M NaCl) was added, which was followed with another incubation for 10 min at 25 °C. This process was terminated with the addition of dinitrosalicylic acid color reagent (1.0 mL), after which the mixture was boiled in water bath for 5 min and subsequently cooled down to room temperature. The reaction mixture was then diluted by adding 10 mL of distilled water, and absorbance measured at 540 nm using the spectrophotometer (Agilent 8453, Agilent Technologies, Waldbronn, Germany). The α-amylase inhibitory activity was expressed as percentage of inhibition (IC_50_). Acarbose was used as a positive control.

### 4.4. Antibacterial and Anticandidal Activity

The following strains of bacteria were used in this study: *Proteus vulgaris* (B44), *Staphylococcus lugdunensis* (B43) and *S. epidermidis* (B45). As for the yeasts, the following species were evaluated: *Candida kefyr* (Y289), *C. krusei* (Y454) and *C. albicans* (Y177). All the microoganisms were deposited at bacterial and fungal unit collection of the Mycological laboratory of the Institute for Biological Research “Siniša Stanković”, National Institute of the Republic of Serbia, University of Belgrade. Evaluated pathogenic microorganisms were clinical samples, isolated from the infected skin [49]. Antibacterial and anticandidal activities of TVH were evaluated using microdilution method in 96-well microtiter plates. The minimum inhibitory concentrations (MIC) and minimal bactericidal/fungicidal concentrations (MBC/MFC) of extracts were determined as described previously [49]. Streptomycin and ketoconazole were used as positive controls.

### 4.5. Animal Protocol

Forty-two healthy male *Wistar albino* rats (six weeks old, body-weight 200 ± 30 g) included in this study were obtained from the Military Medical Academy, Belgrade, Serbia. The animals were housed under controlled regular environmental conditions at room temperature 22 ± 2 °C, with 12 h of automatic illumination daily. The animals were fed with standard rat diet (9% fat, 20% protein, 53% starch and 5% fiber) and tap water *ad libitum*. The experimental design was performed in the laboratory for cardiovascular physiology of the Faculty of Medical Sciences, University of Kragujevac, Serbia in accordance with the current ethical norms approved by the Ethics Committee of the Faculty of Medical Sciences, University of Kragujevac, Kragujevac, Serbia, number: 06/17. All experiments involving animals were conducted in strict accordance with the European Directive for Protection of the Vertebrate Animals used for Experiments (86/609/EEC) and the principles of Good Laboratory Practice (GLP).

After a one-week environment adaptation, MetS was induced by feeding the rats with high-fat diet (HFD—25% fat, 15% protein, 51% starch and 5% fiber) for 4 weeks followed by a single intraperitoneal injection of streptozotocin (STZ) in a dose of 25 mg/kg [50]. MetS was confirmed by measuring fasting glucose and insulin levels, lipid status and blood pressure 72 h post-streptozotocin injection and the animals with fasting blood glucose above 7 mmol/L, insulin above 6 mmol/L, hyperlipidemia (TG, LDL, HDL, TC) and blood pressure above 140/85 mmHg were included in the study

### 4.6. Experimental Design

All rats were divided into the five groups: CTRL (*n* = 8)—untreated healthy animals fed with standard diet; MetS (*n* = 8)—untreated rats with induced MetS; H-TV (*n* = 8)—rats treated with 300 mg/kg (high dose of TV); M-TV (*n* = 8)—rats treated with 200 mg/kg (medium dose of TV); L-TV (*n* = 8)—rats treated with 100 mg/kg (low dose of TV). Groups of animals with induced MetS were fed with HFD till the end of the study. To mimic the most common and the easiest administration route, TVH was administered per os at the appropriate dose every day at the same time for four weeks.

### 4.7. Oral Glucose Tolerance Test (OGTT) and Glucose Levels Determination

After the end of 4-week experimental protocol with TVH, OGTT was conducted. Glucose levels were instantly evaluated by the Accu-Chek (Roche Diagnostics, Indianapolis, IN, USA) glucometer with its comparing strips. Following an overnight fasting, the blood from tail tip of rats were collected to assess the blood glucose levels (0 h of measurement). In addition to oral administration of glucose in a dosage of 2 g/kg of body weight, blood samples were collected at 60 and 120 min after a glucose loading [51].

### 4.8. Insulin Level Measurement

After 4-week protocol, we determined insulin level from serum samples by using ELISA method, according to manufacturer’s instructions (Elabscience Cat. No. E-EL-R3034).

### 4.9. Blood Pressure and Heart Rate Measurement

Using tail-cuff noninvasive method BP system (Rat Tail Cuff Method Blood Pressure Systems (MRBP-R), IITC Life Science Inc., Los Angeles, CA, USA), the values of systolic and diastolic blood pressures (SBP and DBP), as well as heart rate (HR), were measured. In order to confirm the induction of MetS all of these parameters were estimated at the beginning of the experimental period (72 h after STZ injection) as well as at the end of 4-week experimental protocol [52].

### 4.10. Echocardiographic Evaluation

After the four-week protocol, transthoracic echocardiograms were performed in all examined groups, using a Hewlett-Packard Sonos 5500 (Andover, MA, USA) sector scanner equipped with a 15.0-MHz phased-array transducer [53]. Rats were anaesthetized by intraperitoneal injection of a mixture of ketamine (10 mg/kg) and xylazine (5 mg/kg), shaved and, from the parasternal long-axis view in two-dimensional mode, the cursor was positioned perpendicularly to the interventricular septum and posterior wall of the left ventricle (LV) at the level of the papillary muscles and M-mode images were obtained. Interventricular septal wall thickness at end-diastole and at end-systole (IVSd and IVSs), LV internal dimension at end-diastole and at end-systole (LVIDd and LVIDs), LV posterior wall thickness at end-diastole and at end-systole (LVPWd and LVPWs) and fractional shortening (FS) percentage were recorded with M-mode. Ejection fraction (%) was calculated according to Teicholz formula [53].

### 4.11. Lipid Status

Serum samples, collected at the end of protocol, were also used for spectrophotometic determination (Dimension Xpand, Siemens, IL, USA) of lipid status parameters (total cholesterol (TC), triglycerides (TG), high- and low-density lipoprotein (HDL and LDL)).

### 4.12. Systemic Oxidative Stress

After echocardiographic measurement, all animals were sacrificed, and blood samples were collected from jugular vein to evaluate the systemic redox state. Namely, venous blood samples were centrifuged in order to separate the plasma and red blood cells, which were stored at −20 °C until biochemical analysis. The following pro-oxidant parameters were determined from plasma samples: hydrogen peroxide (H_2_O_2_), superoxide anion radical (O_2_^−^), nitrites (NO_2_^−^), and index of lipid peroxidation (TBARS). Parameters of the antioxidative defense system were determined from erythrocyte lysate samples: the activity of superoxide dismutase (SOD) and catalase (CAT) and the level of reduced glutathione (GSH) as previously described [54].

### 4.13. Ex Vivo Cardiac Function

At the moment of sacrificing, rat hearts were quickly isolated, immersed in cold saline and retrogradely perfused on Langendorff apparatus (Langendorff apparatus, Experimetria Ltd., 1062 Budapest, Hungary) with Krebs–Henseleit solution. Retrograde perfusion was performed through the ascending aorta at a constant coronary perfusion pressure of 70 cm H_2_O. Specialized transducer (sensor) was inserted in the left ventricle in order to continuously monitor the following parameters of myocardial function: maximum and minimum rate of left ventricular pressure development (dp/dt max, dp/dt min); systolic and diastolic left ventricular pressure (SLVP and DLVP); and heart rate (HR), while coronary flow (CF) was measured flowmetrically. In order to examine the myocardial function, after the stabilization period at 70 cm H_2_O, the perfusion pressure was gradually decreased to 60 cm H_2_O and then increased to 80, 100 and 120 cm H_2_O (pressure changing protocol 1, PCP 1). Afterwards, the perfusion pressure was decreased to 40 cm H_2_O and the same protocol was repeated, increasing the pressure from 40 to 120 cm H_2_O (pressure changing protocol 2, PCP 2) [55].

### 4.14. Statistical Analysis

IBM SPSS 20.0 for Windows was used for statistical data processing. The Shapiro–Wilk test was used to examine the normality of the distribution. Data are expressed as mean value ± standard deviation and analyzed by one-way analysis of variance (ANOVA) tests, followed by Tukey’s post hoc test for multiple comparisons when distribution between groups was normal. Kruskal–Wallis was used for comparison between groups where the distribution of data was different than normal. A value of *p* < 0.05 was considered statistically significant.

## 5. Conclusions

While various types of cancer take second place as causes of death, cardiovascular diseases and their accompanying conditions remain in this list’s first infamous place as the main leader and cause of most deaths per year on a global scale. This aftermath continuously drives forward research in this area, with in vivo experimental setup being a matter of absolute necessity. Along with diabetes, as a sweet, silent killer that affects more people each year, MetS patients with developed symptoms, as well as those with pre-developed conditions or who are at risk, must be informed of all the changes in lifestyle and possible therapy approaches in a timely manner. Thus, by conducting this type of research, we may develop new strategies to decrease this high proportion of patients and possibly reduce, to some extent, the extremely high costs of their treatment as well as their suffering. One main approach to changing their lifestyle habits which will impact their health status is through changes in the diet. This is the part where mushrooms fit very well, due to presence of numerous compounds with multiple bioactive potential, including polysaccharides, as thorougly investigated in the present study. Along with the fact that the consumption of *T. versicolor* versatile products may be regarded as safe even in cases of prolonged application, it seems that this mushroom is yet to experience its brightest moments.

## Figures and Tables

**Table 1:** FT-IR absorption bands of TVH.

	Absorption (cm^−1^)
Functional Groups	TVH
Stretching vibration of O-H group	3273.18
-CH stretching vibration	2915.27
Bound water	1634.65
-CH (O-CH_2_) stretching vibration	1375.06
C-O stretching vibration	1240.63
β-linked glycosyl group	1029.09

**Table 2:** Monosaccharide composition of the TVH by GC-MS.

No.	Monosaccharide Standards	Rt (min)	TVH (%)
1.	Arabinose	14.05	n.d. *
2.	Rhamnose	14.50	3.34
3.	Fucose	14.75	n.d. *
4.	Xylose	15.19	0.65
5.	Mannose	16.13	n.d. *
6.	Galactose	17.08	37.58
7.	Glucose	17.52	46.01

* n.d: not detected.

**Table 3:** Inhibitory potential of mushroom species on α-amylase linked to type-2 diabetes treatment.

Sample	α-Amylase Inhibition; IC_50_ (µg/mL)
TVH	n.a. ^a^
Acarbose	87.15 ± 2.93

^a^ n.a.—not active at the tested concentrations.

**Table 4:** Antibacterial and anticandidal activity of TVH (mg/mL).

Microorganisms		TVH	Streptomycin/Ketoconazole
*Proteus vulgaris* (B44) (clinical isolate)	MIC	8.00	0.003
	MBC	16.00	0.006
*S. lugdunensisis* (B43) (clinical isolate)	MIC	>16.00	0.003
	MBC	>16.00	0.006
*S. epidermidis* (B45) (clinical isolate)	MIC	8.00	0.10
	MBC	16.00	0.20
*C. kefyr* (Y289) (clinical isolate)	MIC	8.00	0.015
	MBC	16.00	0.030
*C. krusei* (Y454) (clinical isolate)	MIC	8.00	0.015
	MBC	16.00	0.030
*C. albicans* (Y177) (clinical isolate)	MIC	8.00	0.015
	MBC	16.00	0.030

**Table 5:** Effects of TVH administration on echocardiographic parameters of left ventricle.

Group	IVSd (cm)	LVIDd (cm)	LVPWd (cm)	IVSs (cm)	LVIDs (cm)	LVPWs (cm)	FS (%)	EF (%)
*CTRL*	0.174 ± 0.04	0.758 ± 0.15	0.198 ± 0.03	0.234 ± 0.03	0.405 ± 0.08	0.200 ± 0.05	48.836 ± 3.58	82.504 ± 7.85
*MetS*	0.162 ± 0.03	0.670 ± 0.17	0.203 ± 0.02	0.219 ± 0.05	0.444 ± 0.12	0.241 ± 0.03	35.854 ± 8.16 ^a^	67.544 ± 8.97 ^a^
*H-TV*	0.130 ± 0.02 ^ab^	0.531 ± 0.12 ^ab^	0.162 ± 0.0 ^ab^	0.173 ± 0.05 ^ab^	0.304 ± 0.09 ^ab^	0.167 ± 0.05 ^ab^	44.790 ± 7.43 ^b^	79.938 ± 6.03 ^b^
*M-TV*	0.126 ± 0.02 ^ab^	0.611 ± 0.24 ^abc^	0.162 ± 0.04 ^ab^	0.163 ± 0.06 ^ab^	0.346 ± 0.11 ^ab^	0.207 ± 0.09 ^c^	40.241 ± 2.97 ^ab^	78.490 ± 4.43 ^b^
*L-TV*	0.141 ± 0.02 ^ab^	0.536 ± 0.17 ^abd^	0.167 ± 0.03 ^ab^	0.163 ± 0.05 ^ab^	0.371 ± 0.13 ^abc^	0.175 ± 0.04 ^abc^	31.295 ± 4.63 ^acd^	65.271 ± 6.70 ^acd^

IVSd, IVSs—interventricular septal wall thickness at end-systole and end-diastole; LVIDd, LVIDs—left ventricular internal dimension at end-systole and end-diastole; LVPWd, LVPWs—left ventricular posterior wall thickness; FS—fractional shortening; EF—ejection fraction; CTRL—control group of healthy non-treated rats; MetS—control group of rats with induced metabolic syndrome; H-TV—rats with metabolic syndrome treated with high dose of *T. versicolor*; M-TV—rats with metabolic syndrome treated with medium dose of *T. versicolor*; L-TV—rats with metabolic syndrome treated with low dose of *T. versicolor.* Data are presented as means ± standard deviation. Statistical significance at the level *p* < 0.05: ^a^ compared to CTRL; ^b^ compared to MetS; ^c^ compared to H-TV group; ^d^ compared to M-TV group.

## Data Availability

Data is contained within the article.

## Supplementary Materials

The following supporting information can be downloaded at: https://www.mdpi.com/article/10.3390/ph16060787/s1, Figure S1: FT-IR spectrum of TVH. Figure S2: The GC-MS chromatogram of monosaccharide standards. Figure S3: The GC-MS chromatogram of monosaccharide composition of TVH. Figure S4: HPLC chromatogram of TVH.

## References

[1] Saklayen M.G. (2018). The Global Epidemic of the Metabolic Syndrome. Curr. Hypertens Rep..

[2] Alberti K.G., Eckel R.H., Grundy S.M., Zimmet P.Z., Cleeman J.I., Donato K.A., Fruchart J.C., James W.P., Loria C.M., Smith S.C. (2009). International Diabetes Federation Task Force on Epidemiology and Prevention, National Heart, Lung, and Blood Institute, American Heart Association, World Heart Federation, International Atherosclerosis Society, International Association for the Study of Obesity Harmonizing the metabolic syndrome: A joint interim statement of the International Diabetes Federation Task Force on Epidemiology and Prevention; National Heart, Lung, and Blood Institute; American Heart Association; World Heart Federation; International Atherosclerosis Society; and International Association for the Study of Obesity. Circulation.

[3] Pérez-Martínez P., Mikhailidis D.P., Athyros V.G., Bullo M., Couture P., Covas M.I., de Koning L., Delgado-Lista J., Díaz-López A., Drevon C.A. (2017). Lifestyle recommendations for the prevention and management of metabolic syndrome: An international panel recommendation. Nutr. Rev..

[4] Teng J.F., Lee C.H., Hsu T.H., Lo H.C. (2018). Potential activities and mechanisms of extracellular polysaccharopeptides from fermented *Trametes versicolor* on regulating glucose homeostasis in insulin-resistant HepG2 cells. PLoS ONE.

[5] Xu L., Li Y., Dai Y., Peng J. (2018). Natural products for the treatment of type 2 diabetes mellitus: Pharmacology and mechanisms. Pharmacol. Res..

[6] Wińska K., Mączka W., Gabryelska K., Grabarczyk M. (2019). Mushrooms of the Genus Ganoderma Used to Treat Diabetes and Insulin Resistance. Molecules.

[7] Lu Y., Jia Y., Xue Z., Li N., Liu J., Chen H. (2021). Recent Developments in *Inonotus obliquus* (Chaga mushroom) Polysaccharides: Isolation, Structural Characteristics, Biological Activities and Application. Polymers.

[8] Khan M.A., Tania M., Liu R., Rahman M.M. (2013). *Hericium erinaceus*: An edible mushroom with medicinal values. J. Complement. Integr. Med..

[9] Chen C.H., Kang LLo H.C., Hsu T.S., Lin F.Y., Lin Y.S., Wang Z.J., Chen S.T., Shen C.L. (2015). Polysaccharides of *Trametes versicolor* improve bone properties in diabetic rats. J. Agric. Food Chem..

[10] Tel-Çayan G., Çayan F., Deveci E., Duru M.E. (2021). Phenolic profile, antioxidant and cholinesterase inhibitory activities of four *Trametes* species: *T. bicolor*, *T. pubescens*, *T. suaveolens*, and *T. versicolor*. J. Food Meas. Charact..

[11] Meng F., Lin Y., Hu L., Feng W., Su P., Wu L. (2022). The Therapeutic Effect of *Coriolus versicolor* Fruiting Body on STZ-Induced ICR Diabetic Mice. J. Healthc. Eng..

[12] Habtemariam S. (2020). *Trametes versicolor* (Synn. *Coriolus versicolor*) Polysaccharides in Cancer Therapy: Targets and Efficacy. Biomedicines.

[13] Bains A., Chawla P. (2020). In vitro bioactivity, antimicrobial and anti-inflammatory efficacy of modified solvent evaporation assisted *Trametes versicolor* extract. Biotech.

[14] Angelova G., Brazkova M., Mihaylova D., Slavov A., Petkova N., Blazheva D., Deseva I., Gotova I., Dimitrov Z., Krastanov A. (2022). Bioactivity of Biomass and Crude Exopolysaccharides Obtained by Controlled Submerged Cultivation of Medicinal Mushroom. J. Fungi.

[15] Wang K.F., Sui Y., Guo C., Zhao Liu C. (2017). Improved production and antitumor activity of intracellular protein-polysaccharide from *Trametes versicolor* by the quorum sensing molecule-tyrosol. J. Funct. Foods.

[16] Huang Z., Minglong Z., Wang Y., Zhang S., Jiang X. (2020). Extracellular and Intracellular Polysaccharide Extracts of *Trametes versicolor* Improve Lipid Profiles Via Serum Regulation of Lipid-Regulating Enzymes in Hyperlipidemic Mice. Curr. Microbiol..

[17] Zhang X., Zhenyu C., Mao H., Hu P., Li X. (2021). Isolation and structure elucidation of polysaccharides from fruiting bodies of mushroom *Coriolus versicolor* and evaluation of their immunomodulatory effects. Int. J. Biol. Macromol..

[18] Kozarski M., Klaus A., Nikšić M., Vrvić M.M., Todorović N., Jakovljević D., Griensven L.J.L.D.V. (2012). Antioxidative activities and chemical characterization of polysaccharide extracts from the widely used mushrooms *Ganoderma applanatum*, *Ganoderma lucidum*, *Lentinus edodes* and *Trametes versicolor*. J. Food Compos. Anal..

[19] Jhan M.H., Yeh C.H., Tsai C.C., Kao C.T., Chang C.K., Hsieh C.W. (2016). Enhancing the Antioxidant Ability of *Trametes versicolor* Polysaccharopeptides by an Enzymatic Hydrolysis Process. Molecules.

[20] Cui J., Kim K., Goh T., Archer T., Singh H. (2007). Characterisation and bioactivity of protein-bound polysaccharides from submerged-culture fermentation of *Coriolus versicolor* Wr-74 and ATCC-20545 strains. J. Ind. Microbiol. Biotechnol..

[21] Aramabašić Jovanović J., Mihailović M., Uskoković A., Grdović N., Dinić S., Vidaković M. (2021). The Effects of Major Mushroom Bioactive Compounds on Mechanisms That Control Blood Glucose Level. J. Fungi..

[22] Kifle Z.D., Enyew E.F. (2020). Evaluation of *In Vivo* Antidiabetic, *In Vitro* α-Amylase Inhibitory, and *In Vitro* Antioxidant Activity of Leaves Crude Extract and Solvent Fractions of *Bersama abyssinica* Fresen (Melianthaceae). J. Evid. Based Integr. Med..

[23] Ni L., Min C. (2019). Metabolic Syndrome and Skin Disease: Potential Connection and Risk. Int. J. Dermatol..

[24] Shi S., Yin L., Shen X., Dai Y., Wang J., Yin D., Zhang D., Pan X. (2022). β-Glucans from *Trametes versicolor* (L.) Lloyd Is Effective for Prevention of Influenza Virus Infection. Viruses.

[25] Vetter J. (2023). The Mushroom Glucans: Molecules of High Biological and Medicinal Importance. Foods.

[26] Ren Y., Li S., Song Z., Luo Q., Zhang Y., Wang H. (2022). The Regulatory Roles of Polysaccharides and Ferroptosis-Related Phytochemicals in Liver Diseases. Nutrients.

[27] Jiang X., Meng W., Li L., Meng Z., Wang D. (2020). Adjuvant Therapy with Mushroom Polysaccharides for Diabetic Complications. Front. Pharmacol..

[28] Lo H.C., Hsu T.H., Lee C.H. (2020). Extracellular Polysaccharopeptides from Fermented Turkey Tail Medicinal Mushroom, *Trametes versicolor* (Agaricomycetes), Mitigate Oxidative Stress, Hyperglycemia, and Hyperlipidemia in Rats with Type 2 Diabetes Mellitus. Int. J. Med. Mushrooms.

[29] Xu S., Ye B., Dou Y., Hu M., Rong X. (2016). *Coriolus versicolor* polysaccharide regulates inflammatory cytokines expression and ameliorates hyperlipidemia in mice. Acta Sci. Nat. Univ. Nankaiensis.

[30] Zhang S., Lei L., Zhou Y., Ye F., Zhao G. (2022). Roles of mushroom polysaccharides in chronic disease management. J. Integr. Agric..

[31] Chan S.W., Tomlinson B., Chan P., Lam C.W.K. (2021). The beneficial effects of *Ganoderma lucidum* on cardiovascular and metabolic disease risk. Pharm. Biol..

[32] Amirullah N.A., Zainal Abidin N., Abdullah N. (2018). The potential applications of mushrooms against some facets of atherosclerosis: A review. Food Res. Int..

[33] Gao Y., Chen G., Dai X., Ye J., Zhou S. (2004). A phase I/II study of Ling Zhi mushroom *Ganoderma lucidum* (W.Curt.:Fr.) Lloyd (Aphyllophoromycetideae) extract in patients with coronary heart disease. Int. J. Med. Mushr..

[34] Wang Y., Li H., Li Y., Zhao Y., Xiong F., Liu Y., Xue H., Yang Z., Ni S., Sahil A. (2019). *Coriolus versicolor* alleviates diabetic cardiomyopathy by inhibiting cardiac fibrosis and NLRP3 inflammasome activation. Phytother. Res..

[35] Quagliariello V., Basilicata M.G., Pepe G., De Anseris R., Di Mauro A., Scognamiglio G., Palma G., Vestuto V., Buccolo S., Luciano A. (2022). Combination of *Spirulina platensis*, *Ganoderma lucidum* and *Moringa oleifera* Improves Cardiac Functions and Reduces Pro-Inflammatory Biomarkers in Preclinical Models of Short-Term Doxorubicin-Mediated Cardiotoxicity: New Frontiers in Cardioncology. J. Cardiovasc. Dev. Dis..

[36] Xie Y.Z., Yang F., Tan W., Li X., Jiao C., Huang R., Yang B.B. (2016). The anti-cancer components of *Ganoderma lucidum* possesses cardiovascular protective effect by regulating circular RNA expression. Oncoscience.

[37] Janjušević L., Karaman M., Šibul F., Tommonaro G., Iodice C., Jakovljević D., Pejin B. (2017). The lignicolous fungus *Trametes versicolor* (L.) Lloyd (1920): A promising natural source of antiradical and AChE inhibitory agents. J. Enzyme. Inhib. Med. Chem..

[38] Janjušević L., Pejin B., Kaišarević S., Gorjanović S., Pastor F., Tešanović K., Karaman M. (2018). *Trametes versicolor* ethanol extract, a promising candidate for health-promoting food supplement. Nat. Prod. Res..

[39] Moazzem Hossen S.M., Akramul H.T., Shahadat Hossain M., Ahmed Sami S., Uddin Emon N. (2021). Deciphering the CNS anti-depressant, antioxidant and cytotoxic profiling of methanol and aqueous extracts of *Trametes versicolor* and molecular interactions of its phenolic compounds. Saudi J. Biol. Sci..

[40] Kıvrak I., Kivrak S., Karababa E. (2020). Assessment of Bioactive Compounds and Antioxidant Activity of Turkey Tail Medicinal Mushroom *Trametes versicolor* (Agaricomycetes). Int. J. Med. Mushrooms.

[41] Rašeta M., Popović M., Knežević P., Šibul F., Kaišarević S., Karaman M. (2020). Bioactive Phenolic Compounds of Two Medicinal Mushroom Species Trametes versicolor and Stereum subtomentosum as Antioxidant and Antiproliferative Agents. Chem. Biodivers..

[42] Bains A., Chawla P., Kaur S., Najda A., Fogarasi M., Fogarasi S. (2021). Bioactives from Mushroom: Health Attributes and Food Industry Applications. Materials.

[43] Chay W.Y., Tham C.K., Toh H.C., Lim H.Y., Tan C.K., Lim C., Wang W.W., Choo S.P. (2017). *Coriolus versicolor* (Yunzhi) Use as Therapy in Advanced Hepatocellular Carcinoma Patients with Poor Liver Function or Who Are Unfit for Standard Therapy. J. Altern. Complement. Med..

[44] Hsu W.K., Hsu T.H., Lin F.Y., Cheng Y.K., Yang J.P. (2013). Separation, purification, and α-glucosidase inhibition of polysaccharides from *Coriolus versicolor* LH1 mycelia. Carbohydr. Polym..

[45] Liu H., Fan H., Zhang J., Zhang S., Zhao W., Liu T., Wang D. (2020). Isolation, purification, structural characteristic and antioxidative property of polysaccharides from *A. cepa* L. var. agrogatum Don. Food Sci. Hum. Wellness.

[46] Jeff I.B., Li S., Peng X., Kassim M.R.R., Liu B., Zhou Y. (2013). Purification, structural elucidation and antitumor activity of a novel mannogalactoglucan from the fruiting bodies of *Lentinus edodes*. Fitoterapia.

[47] Deveci E., Çayan F., Tel-Çayan G., Duru M.E. (2019). Structural characterization and determination of biological activities for different polysaccharides extracted from tree mushroom species. J. Food Biochem..

[48] Stojkovic D., Smiljkovic M., Ciric A., Glamoclija J., Van Griensven L., Ferreira I.C., Sokovic M. (2019). An insight into antidiabetic properties of six medicinal and edible mushrooms: Inhibition of α-amylase and α-glucosidase linked to type-2 diabetes. S. Afr. J. Bot..

[49] Đorđevski N., Abdullahi I.U., Zengin G., Božunović J., Gašić U., Ristanović E., Ćirić A., Nikolić B., Stojković D. (2023). Chemical and Biological Investigations of *Allium scorodoprasum* L. Flower Extracts. Pharmaceuticals.

[50] Abdel-Hamid H.A., Abdalla M.M.I., Zenhom N.M., Ahmed R.F. (2019). The effect of peptide tyrosine tyrosine (PYY3-36), a selective Y2 receptor agonist on streptozotocin-induced diabetes in albino rats. Endocr. Regul..

[51] Jeremic J.N., Jakovljevic V.L., Zivkovic V.I., Srejovic I.M., Bradic J.V., Milosavljevic I.M., Mitrovic S.L., Jovicic N.U., Bolevich S.B., Svistunov A.A. (2020). Garlic Derived Diallyl Trisulfide in Experimental Metabolic Syndrome: Metabolic Effects and Cardioprotective Role. Int. J. Mol. Sci..

[52] Draginic N.D., Jakovljevic V.L., Jeremic J.N., Srejovic I.M., Andjic M.M., Rankovic M.R., Sretenovic J.Z., Zivkovic V.I., Ljujic B.T., Mitrovic S.L. (2022). *Melissa officinalis* L. Supplementation Provides Cardioprotection in a Rat Model of Experimental Autoimmune Myocarditis. Oxidative Med. Cell. Longev..

[53] Jeremic J.N., Jakovljevic V.L., Zivkovic V.I., Srejovic I.M., Bradic J.V., Bolevich S., Nikolic T.R., Mitrovic S.L., Jovicic N.U., Tyagi S.C. (2019). The cardioprotective effects of diallyl trisulfide on diabetic rats with ex vivo induced ischemia/reperfusion injury. Mol. Cell. Biochem..

[54] Jeremic J., Nikolic Turnic T., Zivkovic V., Jeremic N., Milosavljevic I., Srejovic I., Obrenovic R., Jancic S., Rakocevic M., Matic S. (2018). Vitamin B complex mitigates cardiac dysfunction in high-methionine diet-induced hyperhomocysteinemia. Clin. Exp. Pharmacol. Physiol..

[55] Jakovljevic V., Milic P., Bradic J., Jeremic J., Zivkovic V., Srejovic I., Nikolic Turnic T., Milosavljevic I., Jeremic N., Bolevich S. (2018). Standardized *Aronia melanocarpa* Extract as Novel Supplement against Metabolic Syndrome: A Rat Model. Int. J. Mol. Sci..

