# Supplementary material for: Characterization, In Vitro Biological Activity and In Vivo Cardioprotective Properties of Trametes versicolor (L.:Fr.) Quél. Heteropolysaccharides in a Rat Model of Metabolic Syndrome
Source: Pharmaceuticals (Basel). 2023 May 25;16(6):787. doi: 10.3390/ph16060787 (PMC10301780; doi:10.3390/ph16060787)
Supplement: Supplementary file 1 [file pharmaceuticals-16-00787-s001.zip › pharmaceuticals-2371196-supplementary.pdf]

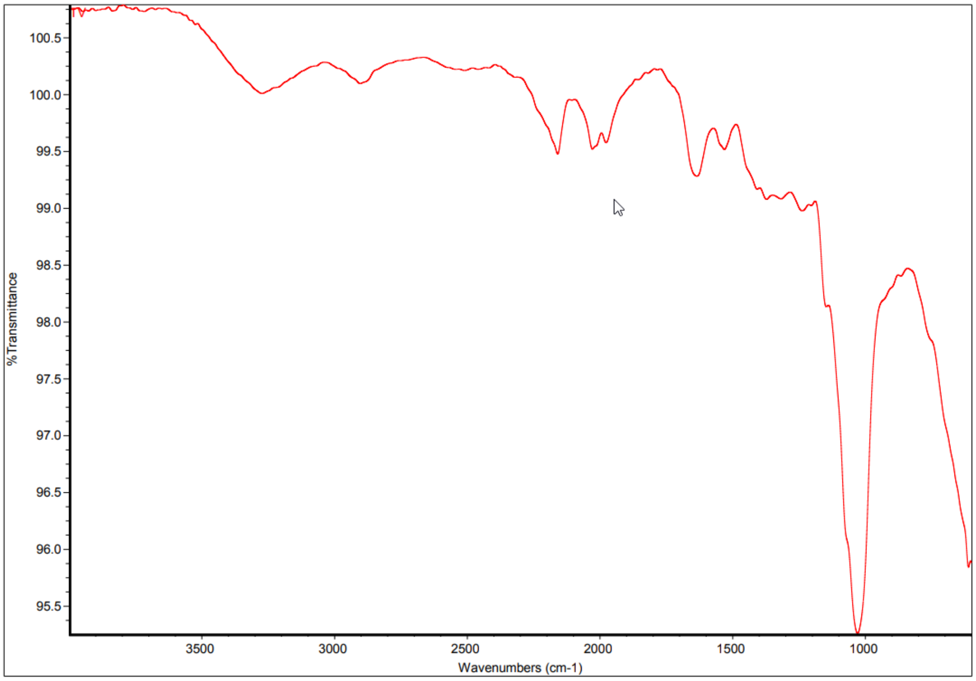

Figure S1. FT-IR spectrum of TVH.

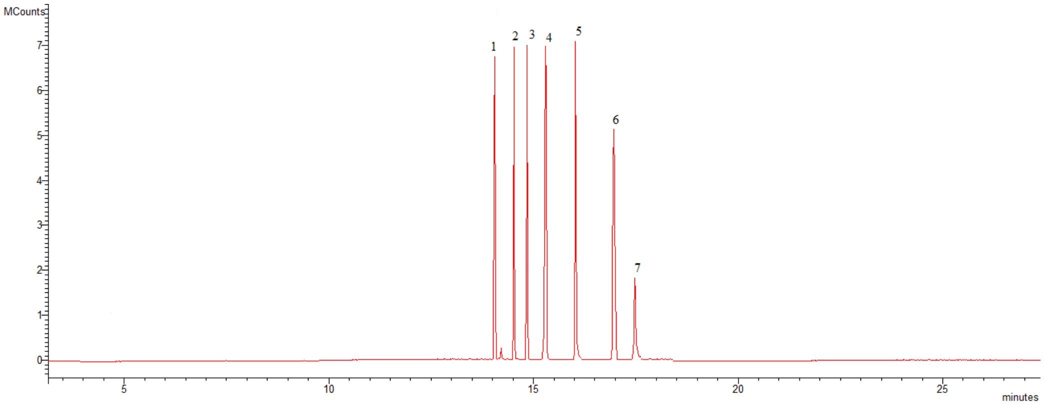

Figure S2. The GC-MS chromatogram of monosaccharide standards.

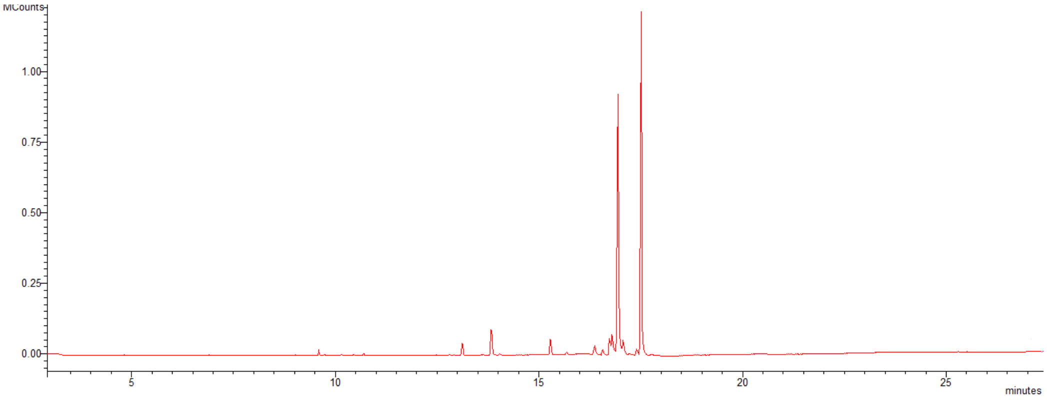

Figure S3. The GC-MS chromatogram of monosaccharide composition of TVH

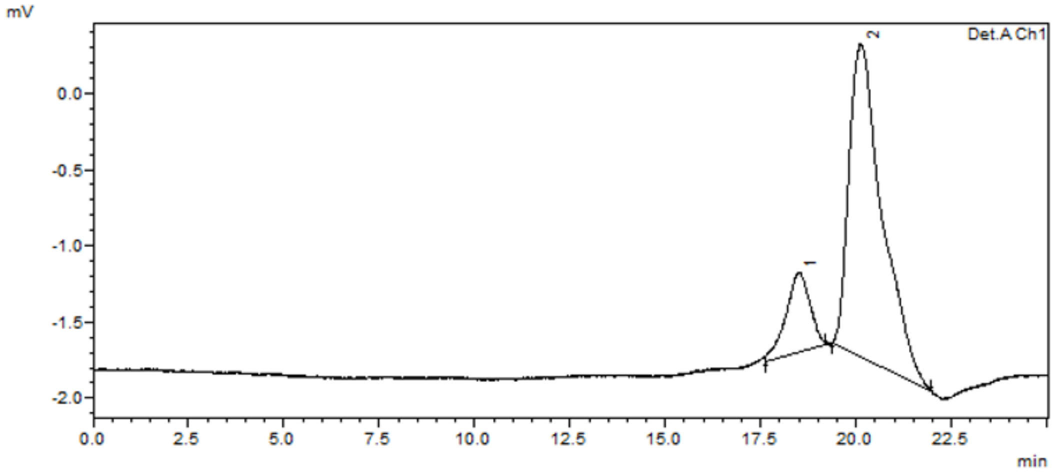

Figure S4. HPLC chromatogram of TVH
